# Replacement of Asymmetric Synaptic Profiles in the Molecular Layer of Dentate Gyrus Following Cycloheximide in the Pilocarpine Model in Rats

**DOI:** 10.3389/fpsyt.2015.00157

**Published:** 2015-11-17

**Authors:** Simone Bittencourt, Luciene Covolan, Clement Hamani, Beatriz M. Longo, Flávio P. Faria, Edna Freymuller, Ole P. Ottersen, Luiz E. Mello

**Affiliations:** ^1^Department of Physiology, Universidade Federal de São Paulo, São Paulo, Brazil; ^2^Division of Neurosurgery, Toronto Western Hospital, Toronto, ON, Canada; ^3^Electron Microscopy Center, Universidade Federal de São Paulo, São Paulo, Brazil; ^4^Department of Anatomy, Centre for Molecular Biology and Neuroscience, University of Oslo, Oslo, Norway

**Keywords:** mossy fiber sprouting, asymmetric synaptic profiles, epilepsy, synaptic plasticity, cycloheximide

## Abstract

Mossy fiber sprouting is among the best-studied forms of post-lesional synaptic plasticity and is regarded by many as contributory to seizures in both humans and animal models of epilepsy. It is not known whether mossy fiber sprouting increases the number of synapses in the molecular layer or merely replaces lost contacts. Using the pilocarpine (Pilo) model of *status epilepticus* to induce mossy fiber sprouting, and cycloheximide (CHX) to block this sprouting, we evaluated at the ultrastructural level the number and type of asymmetric synaptic contacts in the molecular layer of the dentate gyrus. As expected, whereas Pilo-treated rats had dense silver grain deposits in the inner molecular layer (IML) (reflecting mossy fiber sprouting), pilocarpine + cycloheximide (CHX + Pilo)-treated animals did not differ from controls. Both groups of treated rats (Pilo group and CHX + Pilo group) had reduced density of asymmetric synaptic profiles (putative excitatory synaptic contacts), which was greater for CHX-treated animals. For both treated groups, the loss of excitatory synaptic contacts was even greater in the outer molecular layer than in the best-studied IML (in which mossy fiber sprouting occurs). These results indicate that mossy fiber sprouting tends to replace lost synaptic contacts rather than increase the absolute number of contacts. We speculate that the overall result is more consistent with restored rather than with increased excitability.

## Introduction

The functional consequences of mossy fiber sprouting in the epileptic tissue have been interpreted as either contributing to (1–3) or counteracting epileptic seizures (4–6). The controversial nature of this synaptic reorganization has generated at least three hypotheses: the mossy fiber sprouting hypothesis (7) that holds sprouting as a major factor underlying hippocampal hyperexcitability; the dormant basket cell hypothesis (6, 8) that emphasizes the importance of changes in inhibitory activity; and the irritable mossy cell hypothesis (9) that focuses on the hyperexcitability of mossy cells. The apparent inconsistency of the data derives partly from the limited perspective of most anatomical studies, which focus primarily on a single-synaptic input (e.g., sprouted mossy fibers as opposed to data from field neuronal recordings).

Dentate granule cells receive predominant input from the medial septum, entorhinal cortex, and hilus [for review, see Ref. (10)]. In this context, while most studies in this area have concentrated on mossy fiber sprouting, it is clear that synaptic changes from sources other than granule cells might also contribute to the development of epileptogenesis. These, however, remain largely unknown (11) due the lack of staining methods to specifically characterize such terminals (in contrast to the Timm’s staining technique used to study mossy fiber terminals). One approach to address this issue is to study synaptic terminals that innervate the dentate molecular layer at the ultrastructural level.

We have previously demonstrated that induction of *status epilepticus (SE)* in the presence of cycloheximide (CHX) is associated with marked reduction of hilar mossy cell loss in mice and rats (13). Indeed, there is little or no sprouting of mossy fibers (granule cell axons) in animals subject to *SE* under the presence of CHX (14–16). Here, we investigated whether mossy fiber sprouting, and additional synaptic reorganization of the dentate molecular layer, would be associated with an increase in the number of asymmetric synaptic profiles, or simply with synaptic replacement.

## Materials and Methods

### Animals and Protocol for Pilocarpine Induction of Chronic Seizures

All experimental protocols were approved by the Animal Care and Use Ethics Committee of UNIFESP and were performed in accordance with the Society for Neuroscience guidelines for animal research. Male Wistar rats (*n* = 30, 200–250 g) were kept on standard light/dark cycle (12/12 h) with lights on at 7:00 a.m. Animals had free access to rat chow pellets (Nuvilab) and tap water. Seizures were induced by injections of pilocarpine hydrochloride (Pilo, 320 mg/kg, i.p. Merck). Scopolamine methyl bromide (1 mg/kg, i.p., Sigma) was administered 30 min prior to Pilo to reduce its peripheral effects. In addition, one group of animals also received CHX (1 mg/kg, i.p., Sigma) 30 min prior to Pilo (CHX + Pilo). All animals developing *SE* received thionembutal (25 mg/kg, i.p., Cristalia, Brazil) 90 min later, as previously described (17). Four months after *SE*, animals were sacrificed and had their brains processed, as described below.

### Tissue Processing

Two different protocols were performed 120 days after *SE* induction (*Experiment 1* and *Experiment 2*); for each protocol, we analyzed five animals per group. For *Experiment 1*, five animals from each group (Pilo, CHX + Pilo, and control) were transcardially perfused with 500 mL sulfide solution (4% glutaraldehyde, 0.1% Na_2_S, 0.002% CaCl_2_, in 0.12M Millonig’s phosphate buffer, pH7.3) (18). One hour later, brains were processed according to the Timm’s staining method for the ultrastructural evaluation of silver grains in synaptic terminals of the hippocampal dentate molecular layer. After removal from the skull, brains were placed in the same fixative solution for 24 h at 4°C. Coronal sections (100 μm thick) were cut on a vibratome (Vibratome Series 1000 Sectioning System) and transferred to a fresh developing solution (60 mL gum Arabic 50%, 10 mL of a 2M citrate buffer; 15 mL hydroquinone 5.67%, and 15 mL silver lactate 0.73%) for 1.5 h in the dark, under constant agitation, and subsequently processed for electron microscopy (EM).

Another set of five animals for each group (Pilo, CHX + Pilo, and control) was used to evaluate synaptic profiles in the dentate gyrus molecular layer (*Experiment 2*). Under deep anesthesia with thionembutal (50 mg/kg, i.p.), rats were transcardially perfused with 500 mL of modified Karnovsky solution at 4°C (2.5% glutaraldehyde, 2% formaldehyde in 0.1M phosphate buffer, pH 7.4), for at least 1 h. One hour later, brains were removed from the skull, immersed in the same fixative solution for at (19) least 24 h at 4°C, and subsequently processed for EM.

### Electron Microscopy Procedures

Tissue specimens were obtained from the right dorsal hippocampus (corresponding approximately to levels 28–31 of Swanson’s rat brain atlas) (20). Samples remained overnight in a 0.1M pH 7.4 cacodylate buffer solution [Na[CH_3_]^2^.AsO_2_.3H_2_O], at 4°C. After rinsing, specimens were postfixed with 1% OsO_4_ sodium cacodylate buffer, washed in sodium cacodylate buffer, and kept overnight in uranyl acetate. The specimens were then dehydrated in a series of ethanol baths, placed in propylene oxide, transferred to pure Epon resin, and placed in vacuum for 4 h. The block polymerization took place at 60°C for at least 2 days. Seventy-nonometer-thick (silver interference color) and 90-nm-thick (gold interference color) sections were cut (Ultracut S/FC S, Reichert) for *experiments* 2 and 1, respectively. These sections were then stained with 2% uranyl acetate and lead citrate.

#### Methodological Considerations

**Figure 1:**
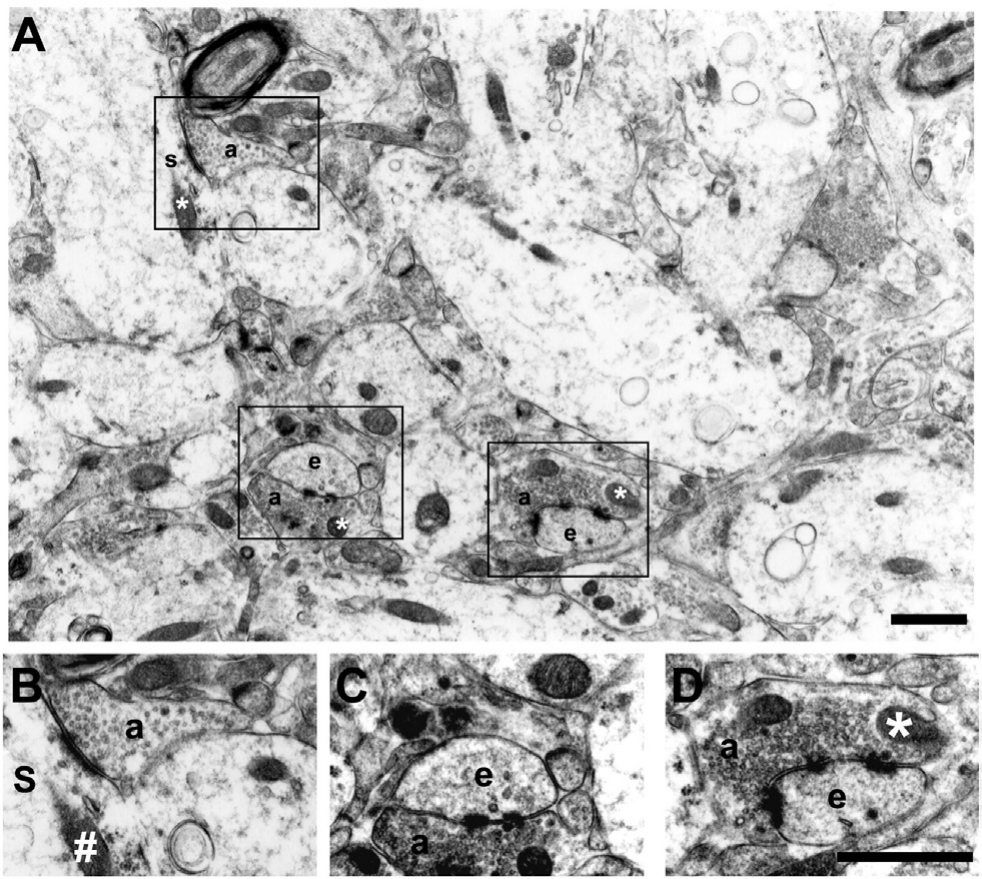
**Electron micrograph of the inner molecular layer showing asymmetric synaptic profiles and their localization in dendritic spines or shafts (A)**. **(B–D)** are higher magnification views of different synaptic contacts: PSD1 [**(B)** – non-perforated type 1, synaptic profiles with a single-synaptic bouton]; PSD2 [**(C)** – perforated type 2, synaptic profiles with two postsynaptic densities]; and PSD3 [**(D)** – perforated type 3, synaptic profiles with more than two postsynaptic densities]. Note the spherical presynaptic vesicles and mitochondrion (*) in the axon terminal (a) contacting a dendritic spine (e) and a dendritic shaft (s) with a mitochondria (^#^). Scale bars, 1.25 μm.

The criteria used to identify ultrastructural synaptic profiles have been previously described (21, 22). Briefly, synaptic profiles were identified by cleft material between parallel membranes of a presynaptic element using at least two spherical vesicles and a postsynaptic element with a postsynaptic density (PSD). Active zones were distinguished from puncta adhaerentia by the lack of a pronounced presynaptic thickening and the usual presence of apposed presynaptic vesicles. We restricted our evaluation to asymmetric synaptic profiles (excitatory synapses) of the dentate molecular layer. The asymmetric synaptic profiles were classified as perforated and non-perforated based on shape of the PSD (23–25). These were categorized as: PSD1 (non-perforated type 1 synapses), synaptic profiles with a single-synaptic bouton associated to a continuous disk shape; PSD2 or PSD3 (perforated types 2 or 3 synapses), synaptic profiles with two or more PSDs, respectively. We have also evaluated the number of synaptic profiles located on dendritic spines versus those located on dendritic shafts. Dendritic spines were discerned from dendritic shafts by morphological features of spines. As an example, the dendritic shaft cytoplasm contains microtubules, mitochondria, and a multivesicular body, while the cytoplasm of the spine consists of stacks of smooth endoplasmic reticulum interdigitated by electron-dense plates (26, 27) (Figure 1).

The number of non-perforated and perforated synapses is not shown in absolute values because a quantification of this order would require the analysis of serial sections. The basic assumption for counting synaptic profiles at various single sections is the probability that different planes of section would be equally distributed across groups.

The area of silver grain electron-dense deposits from the Timm’s reaction was evaluated by quantitative densitometric stereological analysis, through a cross test system, in approximately the same area of that used for counting the asymmetric synaptic profiles (approximately 1,713 μm^2^ per layer per animal). Although one could consider desirable to obtain quantitative estimates of the relative number of silver grain-containing sprouted fibers, technical limitations in applying the Timm’s method at an ultrastructural level make such estimates potentially unreliable. Moreover, the size and density of silver grains is not linearly related to the concentration of zinc within a terminal. It is important to emphasize that data obtained with Timm’s staining for EM did not allow a clear definition of synaptic membranes. In some of the sections, however, we could observe silver grain clusters around dendritic shafts (putative mossy fiber sprouting terminals) with a non-perforated asymmetric synaptic profile (Figure 1). This is in agreement with a previous study using Timm’s staining in kainate-treated rats (28).

#### Analysis of Synaptic Profiles

In each experiment, six randomly selected photographs were taken from every examined tissue field [in the inner molecular layer (IML) and outer molecular layer (OML)]. Each 285 μm^2^ field was photographed at 5000× and amplified to 15,000×. Quantification of synaptic profiles was performed only in non-overlapping areas (free of large blood vessels, tissue tears, or folds), by counting all asymmetric synaptic profiles in an area of 1,712.88 μm^2^ per dentate molecular layer/animal. Synaptic profiles on the exclusion lines were not counted. The IML and OML boundaries were determined for each section. The rat dentate molecular layer has a 250 μm thickness on average (10). The innermost 50–70 μm are often considered the IML, whereas the outermost 150–200 μm comprise the OML. While this subdivision tends to ignore the intermediate molecular layer, it provides a well-defined distinction between the IML and OML with no chance of sampling overlapping fields. In addition, the characterization of the connectivity of the intermediate molecular layer has always been demonstrated as being similar to that of the OML. Bearing this in mind, we chose to describe our data as a fraction of the afferent connections, dividing the dentate gyrus molecular layer in inner and outer counterparts.

Comparative width analysis of the dentate molecular layer (from dentate granule cell layer to hippocampal fissure) using 100-μm-thick coronal sections revealed that this layer had a similar width in all experimental groups, indicating that there was no differential tissue shrinkage in controls, Pilo-, and CHX + Pilo-treated animals.

The estimation of the area of silver grains-impregnation and that of the density of asymmetric synaptic profiles were determined with a stereological test system method and an unbiased counting frame, respectively (29). The test system was applied as a mask over the final enlarged electron micrograph prints for estimating the area of silver grains. The space between the points of the test system was 11 mm, corresponding to a tissue area of 0.85 μm^2^ (0.92 μm × 0.92 μm).

### Statistical Analysis

Results are presented as mean ± SEM. Comparisons between parameters were carried out by one-way analysis of variance (ANOVA) followed by Newman–Keuls *post hoc* test, using the Statistica 7 software. Significance was set at *P* < 0.05.

## Results

Spontaneous recurrent epileptic seizures were first observed at approximately 15–21 days after *SE* in all animals. However, given that we did not perform a complete 24/7 seizure assessment, it is possible that spontaneous seizures may have emerged earlier.

The comparative width analysis of the dentate molecular layer (from dentate granule cell layer to hippocampal fissure) using 100-μm-thick coronal sections revealed that this layer had a similar width in all experimental groups, indicating that there was no differential tissue shrinkage in control, Pilo-, and CHX + Pilo-treated animals.

### CHX + Pilo Treatment Reduced Deposits of Silver Grains in the IML

**Figure 2:**
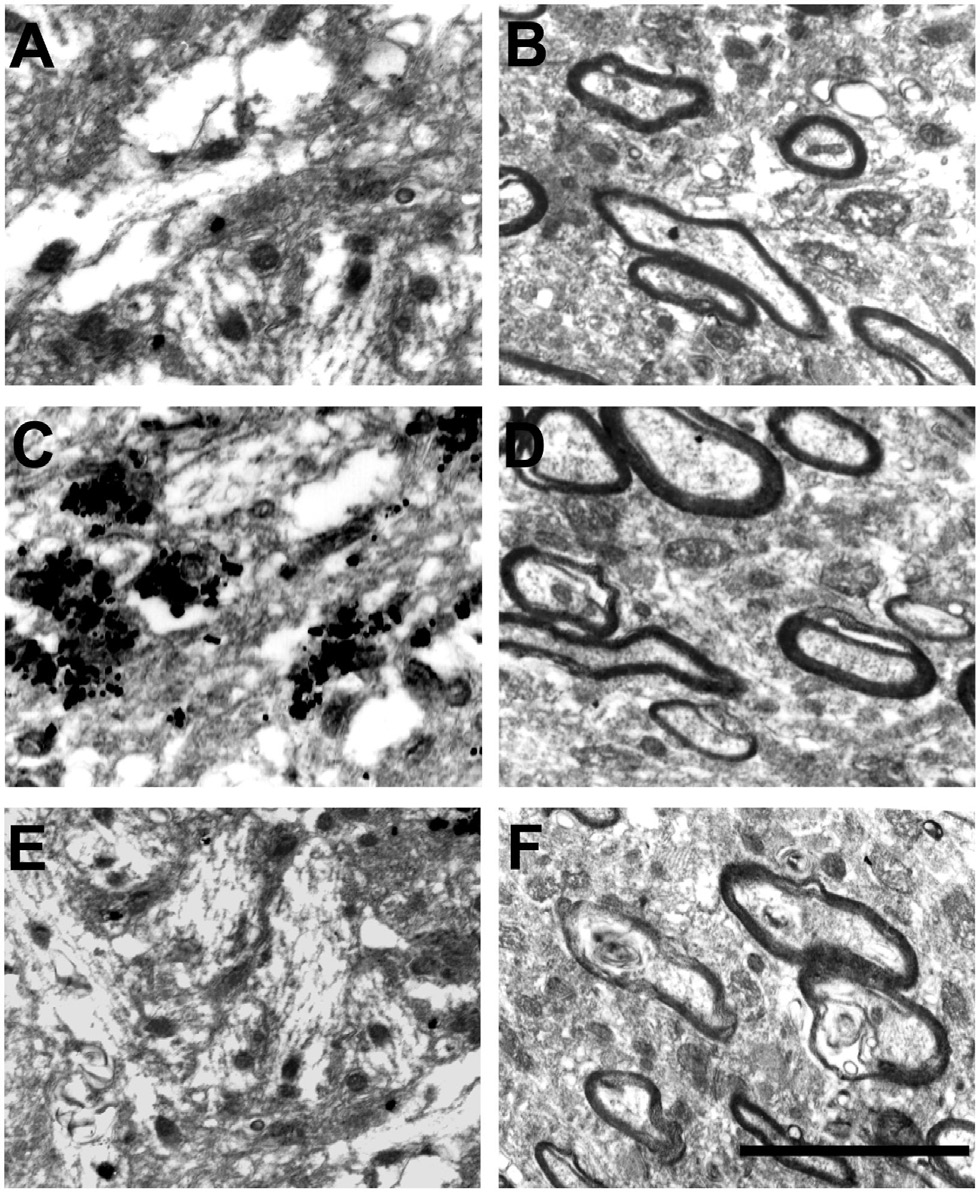
**Electron micrographs of the dentate gyrus molecular layer in controls (A,B), Pilo-treated (C,D) and CHX **+** Pilo-treated animals (E,F)**. **(A,C,E)** represent the inner molecular layer (IML). **(B,D,F)** represent the outer molecular layer (OML). Silver grain dots in the IML were only observed in Pilo-treated animals **(C)**. These profiles have not been found in the outer molecular layer of any of the groups **(B,D,F)**. Scale bar, 150 nm.

**Figure 3:**
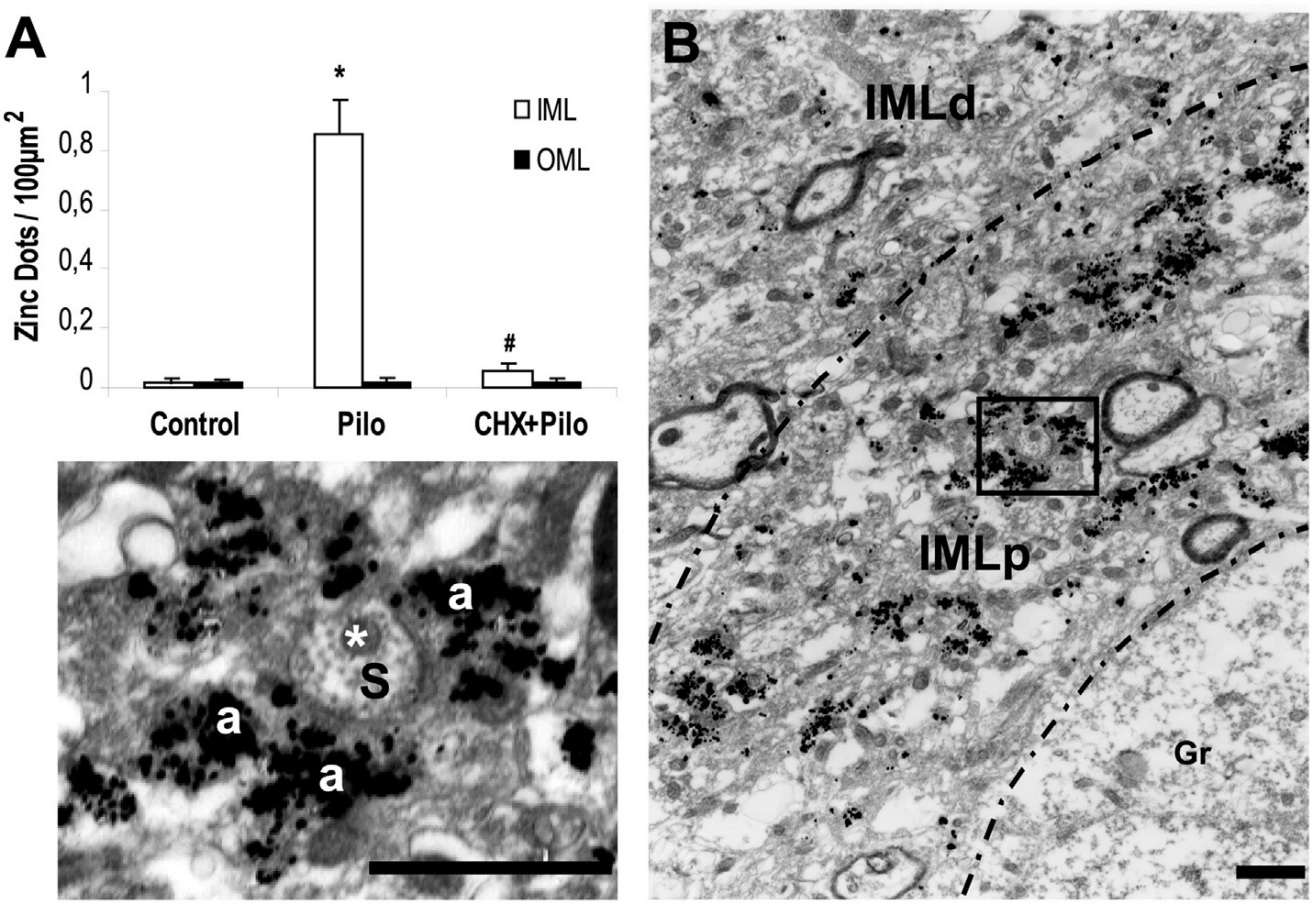
**(A)** Density of silver grains staining per 100 μm^2^ within dentate molecular layer of control, pilocarpine (Pilo)- and cycloheximide + pilocarpine (CHX + Pilo)-treated animals. IML, inner molecular layer; OML, outer molecular layer. *P < 0.001; compared to controls; ^#^*P* < 0.001; compared to the Pilo group. **(B)** Sections at the level of the inner molecular layer staining for mossy fiber sprouting of Pilo-treated animal. Note the greater silver grains staining in the molecular layer more proximal (IMLp) to the granule cell layer (Gr) as compared to the more distal portion of the inner molecular layer (IMLd). In these higher magnification views, histochemically reactive silver grains could easily be localized on the asymmetric synapse contacts. Axon terminal (a) and dendritic shaft (s) with mitochondria (*). IMLp, Inner molecular layer proximal; IMLd, inner molecular layer distal. Scale bars, 1.25 μm.

At the EM level, the Timm’s sulfide silver method did not reveal any silver grains outside the hilus in the dentate gyrus of control animals (Figures 2A and 3A). This finding is in agreement with previous light (13, 14, 30–32) and EM studies (28, 33, 34). By contrast, all Pilo-treated animals had silver grains in the IML but not in the OML (Figures 2C,D, respectively). A detailed examination of the density of silver grains indicated that the higher staining score mainly stemmed from those IML areas closer to the granule cell layer (up to 10 μm apart from the granule cells, Figure 3B), where it was about twice as high as the one recorded in more distant IML regions (50–70 μm from the granule cell layer, Figure 3B). In the IML of Pilo group, silver grains were mostly deposited on terminal axons where asymmetric synaptic profiles could be identified (Figure 3B). In CHX + Pilo-treated animals, the density of silver grains occupying the IML was only 7% of that observed in Pilo-treated animals and similar to that found in the control group (Figures 2A and 2E respectively). In the hilus, the intensity of silver grains labeling did not differ among the three groups of animals. No animal, irrespective of group, showed silver grains in the OML (Figures 2B,D,F).

### Reduced Density of Asymmetric Synaptic Profiles in Epileptic Rats

**Figure 4:**
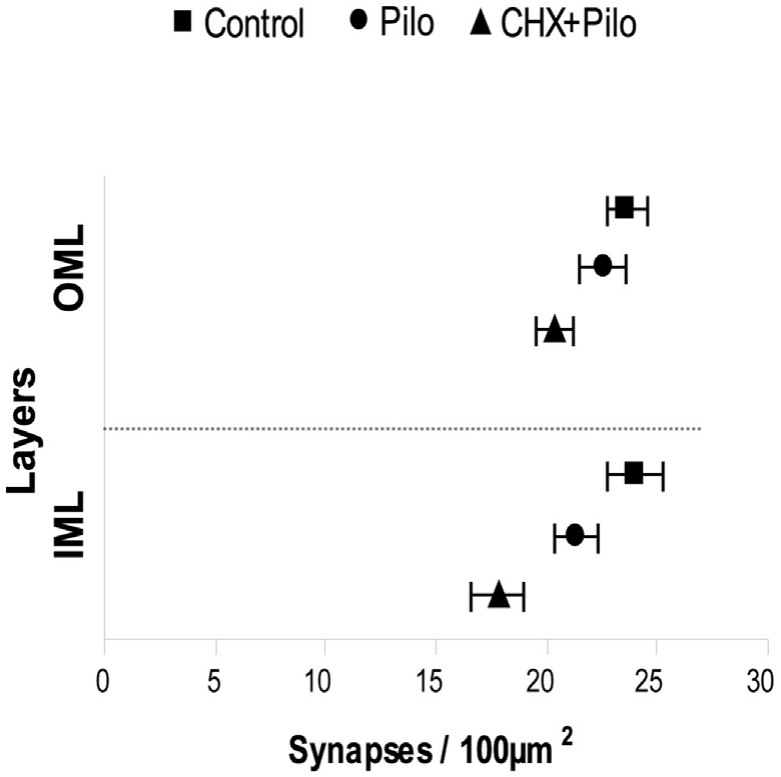
**Means and 95% confidence interval of the total number of synapses obtained using six photomicrographs per layer (IML and OML) in five animals per group**. IML, inner molecular layer; OML, outer molecular layer.

The analysis of asymmetric synaptic profiles in the dentate molecular layer (IML + OML) of the control group revealed a density of 23.64 ± 0.59/100 μm^2^. This was significantly reduced by 8 and 20% in Pilo and CHX + Pilo groups, respectively. It is noteworthy that when considering only the IML, Pilo-treated animals had a synaptic density similar to that of controls (−4.5%). By contrast, animals in the CHX + Pilo group had significant loss of synaptic profiles when compared to control (−14%) and Pilo (–9.5%) groups. Figure 4 summarizes results from Table 1. In the OML, a significant loss in the density of asymmetric synaptic profiles was found in both Pilo (–11%) and CHX + Pilo (–26%) treated rats, as compared with controls. These data suggest that the influence of CHX to inhibit the growth and/or formation of new asymmetric synaptic contacts after Pilo treatment occurs particularly in the OML, as the loss of asymmetric synaptic profiles in this region was twice as high as that observed in the IML.

**Table 1:** **Mean number of asymmetric synaptic profiles per 100 **μ**m^2^ in different layers of the dentate gyrus**.

Type of synapses	Groups	Molecular layer	
		Inner	Outer
PSD 1	Control	21.43 ± 0.66	21.29 ± 0.98
	Pilo	20.38 ± 0.79	19.52 ± 0.66
	CHX + Pilo	18.54 ± 0.62**	15.16 ± 0.86^***,##^
PSD 2	Control	1.67 ± 0.19	2.17 ± 0.21
	Pilo	1.74 ± 0.17	1.32 ± 0.19**
	CHX + Pilo	1.40 ± 0.15	2.18 ± 0.26^#^
PSD 3	Control	0.36 ± 0.07	0.37 ± 0.07
	Pilo	0.29 ± 0.06	0.37 ± 0.09
	CHX + Pilo	0.25 ± 0.04	0.35 ± 0.08

*Data expressed as mean ± SEM. ANOVA followed by Newman–Keuls*.

**P < 0.01, and ***P < 0.001 as compared to controls.

*^#^*P* < 0.05 and ^##^*P* < 0.01 as compared to Pilo group*.

*Each group was comprised of five animals; for each animal, six slices were analyzed*.

### Distribution of PSD1, PSD2, and PSD3 Asymmetric Synaptic Profiles in the Dentate Molecular Layer

In all groups (Pilo, Pilo + CHX, and controls), PSD1 was the most abundant contact type, followed by PSD2 and PSD3. The control group had a mean of 21.36 ± 0.58 PSD1; 1.92 ± 0.14 PSD2, and 0.37 ± 0.05 PSD3 synaptic profiles/100 μm^2^, respectively. This corresponded to 90, 8, and 2% of the asymmetric synaptic profiles found in the dentate molecular layer (Table 1). A similar distribution of synaptic profiles was also seen in Pilo- and CHX + Pilo-treated animals, though with lower absolute values. Significant reductions in the number of PSD1 (21%, *P* < 0.001) were recorded in the CHX + Pilo group as compared to control group. In the same group, we found that the densities of PSD1 were diminished in the IML (*P* < 0.01, as compared to controls) and in the OML (*P* < 0.001 and *P* < 0.01, as compared to control and Pilo groups, respectively). Significant reductions in the number of PSD2 were found in the Pilo group (20%, *P* < 0.05 compared to controls), particularly in the OML (*P* < 0.01, as compared to both controls or CHX + Pilo-treated rats). By contrast, no differences were found in the PSD3 counts across groups (see Table 1). In summary, Pilo-treated animals had a lower density of PSD2 profiles in the OML, whereas the CHX + Pilo group had less PSD1 in both the IML and OML as compared to control group.

### Synaptic Reorganization Preferentially Involved Spine Synapses Rather than Reorganizations on Shaft Synapses

Irrespective of the treatment (control, Pilo-, or CHX + Pilo-treated animals), synapses in the dentate molecular layer were largely located on dendritic spines rather than shafts (Table 2), as previously reported (35, 36). In control animals, 87, 7, and 1% of the synapses in the molecular layer were PSD1, PSD2, and PSD3, respectively. The remaining 5% of synapses were located on dendritic shafts. While a similar frequency was recorded in the Pilo-treated group, this was not the case for rats given CHX + Pilo, which had a relatively lower frequency of PSD1 and a higher number of PSD2 profiles as compared to the other groups (Table 2).

**Table 2:** **Frequency of dendritic spines and dendritic shafts (PSD1, 2, and 3) in the dentate molecular layer**.

Groups	Type of Synapses	IML		OML		Total of Asymmetric Synaptic Profiles (to each group)
*n* = 5		Spines	Shafts	Spines	Shafts	
Control	PSD1	618 (44%)	26 (1.8	606 (43%)	34 (2.4%)	1284/1421 (90.4%)
Pilo		565 (43%)	48 (3.7%)***	545 (42%)	42 (3.2%)*	1200/1312 (91.5%)
CHX + Pilo		519 (46%)	38 (3.3%)***	428 (38%)^**,##^	27 (2.4%)^#^	1012/1138 (88.9%)
Control	PSD2	47 (3.3%)	3 (0.2%)	58 (4.1%)	7 (0.5%)	115/1421 (8.1%)
Pilo		48 (3.7%)	5 (0.4%)	36 (2.7%)**	4 (0.3%)	93/1312 (7.1%)
CHX + Pilo		39 (3.4%)	3 (0.3%)	62 (5.5%)^**,###^	4 (0.4%)	108/1138 (9.5%)
Control	PSD3	11 (0.8%)	0 (0%)	8 (0.6%)	3 (0.2%)	22/1421 (1.6%)
Pilo		7 (0.5%)	1 (0.1%)*	10 (0.8%)	1 (0.1%)	19/1312 (1.5%)
CHX + Pilo		6 (0.5%)	2 (0.2%)*	8 (0.7%)	2 (0.2%)	18/1138 (1.6%)

*Data expressed as frequency of synaptic profiles. Chi-square*.

*P < 0.05, **P < 0.01, and ***P < 0.001 as compared to controls.

*^#^*P* < 0.05, ^##^*P* < 0.01, and ^###^*P* < 0.001 as compared to Pilo group*.

*Each group was comprised of five animals; for each animal, six slices were analyzed*.

In the IML of control animals, 96% of the asymmetric synaptic profiles contacted dendritic spines, while corresponding values for Pilo and CHX + Pilo animals were 92 and 93%, respectively. In the OML, 94, 93, and 94% of the asymmetric synaptic profiles occurred on dendritic spines of control, Pilo, and CHX + Pilo animals, respectively (Table 2). In the IML, the frequency of PSD1 and PSD3 asymmetric synaptic profiles on dendritic shafts of epileptic animals (Pilo and CHX–Pilo groups) was greater than controls (*P* < 0.001 and *P* < 0.05, respectively). In the OML, the frequency of PSD2 asymmetric synaptic profiles in Pilo group was significantly lower than in controls (*P* < 0.01). In CHX + PILO group, while the frequency of PSD1 asymmetric synaptic profiles was significantly reduced (*P* < 0.01), PDS2 synaptic profiles were significantly increased (*P* < 0.01) as compared to controls.

Thus, while control animals lacked PSD3 profiles on dendritic shafts in the IML, these could be seen in both epileptic groups (Pilo and CHX + Pilo) (Table 2). Moreover, for Pilo-treated animals, PSD1 profiles apposing dendritic shafts in the OML were more numerous (*P* < 0.05) than in controls. By contrast, however, CHX–Pilo-treated animals had less PSD1 (*P* < 0.05) in the OML than Pilo-treated animals (Table 1).

**Figure 5:**
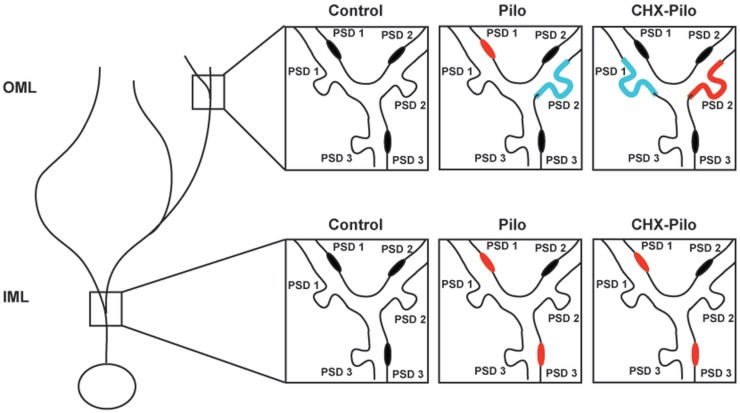
**Schematic view of the changes in the synaptic profiles of the different experimental groups as compared to controls**. Synaptic profiles located on the shaft or spine of dendrites in inner (IML) or outer (OML) molecular layer were identified as PSD1, PSD2, or PSD3. Red denotes type and location of synaptic profile showing a significant increase in density as compared to control animals. Blue represents synaptic types and location that were significantly decreased as compared to control animals. Pilo – animals subjected to pilocarpine-induced *status epilepticus*. CHX + Pilo – animals subjected to pilocarpine-induced *status epilepticus* and co-injected with cycloheximide.

Figure 5 summarizes the significant results of synaptic profiles and dendritic location. In general, the most conspicuous result of our study was that the density of all types of asymmetric synaptic profiles in the epileptic groups was remarkably similar to that registered in controls. There were, however, a few noticeable differences. In Pilo animals, PSD1 contacts were distributed in both IML and OML dendritic shafts, whereas PSD3 contacts were only observed in the IML dendritic shafts. In CHX-treated animals, PSD1 and PSD3 contacts were distributed in IML dendritic shafts, whereas PSD2 contacts were only observed in OML dendritic spines. Finally, comparison of the Pilo and CHX–Pilo groups showed that the latter had a decrease in PSD1 (in both spines and shafts) and an increase in PSD2 (in spines only) in the OML.

## Discussion

The main findings of our study are the following: (1) the density of silver grains in the IML of animals receiving CHX + Pilo was much reduced when compared to that recorded in rats given Pilo alone. (2) CHX + Pilo treatment led to a significant reduction in the density of asymmetric synaptic profiles in the IML and OML (14 and 26%, respectively), whereas animals treated with Pilo did not differ from controls (4.5% for IML and 11% for OML). (3) Both Pilo and CHX + Pilo had an altered distribution of asymmetric synaptic profiles types (e.g., PSD1, PSD2, PSD3) in the dentate molecular layer as compared to controls. The current estimate of 96% of asymmetric synaptic profiles apposing dendritic spines in the IML of control animals is in agreement with previous findings from Buckmaster and colleagues (37). Overall, these findings support and expand our previous observations, suggesting that CHX blocks *SE*-induced supragranular mossy fiber sprouting (14, 15, 38). Indeed, here we not only showed a dramatic reduction of putative Timm-stained mossy fiber terminals in the IML of CHX + Pilo-treated animals but also a reduction in the number of asymmetric synaptic profiles in the IML and OML, and a shift in the type of synaptic terminals present in the same area. The assembly of synaptic profiles with different synaptic efficacies in Pilo- and CHX + Pilo-treated animals may significantly affect information processing in the dentate gyrus.

### Silver Grain Deposits and Asymmetric Synaptic Profiles in the Dentate Molecular Layer

The *SE*-related hilar cell loss in the Pilo model is intense (17, 39) and has been considered to be critical for the development of subsequent mossy fiber sprouting given that hilar neurons represent 36% of all inputs to the dentate IML (40). Our current demonstration of a similar number of IML asymmetric synaptic profiles in both control and Pilo-treated animals further indicates that the synaptic reorganization observed in mossy fiber sprouting represents a tendency to replace lost synaptic contacts rather than the establishment of additional synaptic contacts. In the CHX + Pilo group, we did not find a correspondent loss of asymmetric synaptic profiles, despite the 93% reduction in Timm’s IML labeling. Considering that CHX inhibited the mossy fiber sprouting from hilus, the similarity in the number of asymmetric synaptic profiles between control and CHX + Pilo animals could be an indication of synaptic plasticity from other sources, such as entorhinal cortex, given that this structure is a major source of afferent projections to the dentate gyrus (41). On the other hand, CHX may have protected the hilus from damage, given that the loss of hilar (as usually suggested) and entorhinal neurons are often a pre-requisite for mossy fibers to sprout in animal model of epilepsy, including Pilo (39, 42, 43). In fact, neuronal loss in the hilus (44) and entorhinal cortex (unpublished data) is less intense in CHX + Pilo than in Pilo animals. However, the present study is not sufficient to specifically elucidate whether the effects stem from CHX-related cell protection in the hilus, entorhinal cortex, or both. Our own previous findings in Pilo-treated animals provided evidence indicative of a protective role of CHX over hilar mossy cells in mice (12) and rats (13).

### Synaptic Profile Morphology

Ganeshina and collaborators (2004) demonstrated that perforated synapses (PSD2 and PSD3) have an invariably higher concentration of AMPA receptors than non-perforated synapses (~660% more) and have 80% more immunoreactive NMDA receptors than non-perforated synapses (PSD1) in the hippocampal CA1 stratum radiatum. Moreover, ~35% of non-perforated synapses do not show any immunoreactivity for AMPA receptors (45), and may thus be considered “silent” synapses (46, 47). Therefore, perforated synapses may evoke synaptic responses with AMPA and NMDA receptors-mediated components of an exceptional magnitude and thereby contribute to an enhancement of synaptic transmission.

It is widely accepted that perforated (PSD2 and PSD3) synapses are much more efficient in impulse transmission than non-perforated (PSD1) synapses (45, 48–55). In the present work, the decreased number of PSD1 in the IML and OML of the CHX + Pilo group may suggest a reduced excitability. To the same extent, however, only Pilo-treated animals had a significant loss of the more effective PSD2 synaptic contacts (39% less compared to control group) in the OML (Table 1). Thus, the reduction in the number of perforated synapses (PSD2) in Pilo animals might have a greater impact in reducing the excitability of the OML than the reduction of non-perforated synapses in CHX + Pilo animals.

Interpreting changes in synaptic morphology is not an easy task. The total number of PSD1 in our study was one order of magnitude greater than that of PSD2 and two- to fivefold greater than that of PSD3. Therefore, a small change of 10% in the frequency of PSD1 synapses could indicate a decrease or increase of 150 synapses. By contrast, changes in only 15 PSD2 synapses may result in a similar 10% increase or decrease in the number of synapses. Of course, given that we did not perform any functional evaluation in the current study, these are merely assumptions based on anatomical data.

The reduction of silver grain profiles seen at the ultrastructural level in the IML of CHX + Pilo-treated animals, as compared to Pilo-treated animals, represents important additional evidence of CHX ability to block mossy fiber sprouting, confirming our previous findings (14, 16) despite contrasting data (56, 57). Our results suggest that the dentate molecular layer synaptic reorganization that follows *SE* is a fine tuned process, which might be more suitable to restore dentate function than increasing excitation.

## Author Contributions

SB manipulated animals and performed the EM procedures, discussed results, and wrote the first draft of the manuscript; FF and EF helped in the EM procedures, CH, BL, and OO discussed results and commented on discussion. LC and LM conceived the experimental design, participated in discussion of results and the final writing of the manuscript. All authors read and approved the final manuscript.

## Conflict of Interest Statement

The authors declare that the research was conducted in the absence of any commercial or financial relationships that could be construed as a potential conflict of interest.
